# Plant-Derived Monoclonal Antibody Against Botulinum Neurotoxin: Pharmacokinetic Evaluation in a Mouse Model

**DOI:** 10.3390/ph19101597

**Published:** 2026-10-09

**Authors:** Nipaporn Simsom, Pipob Suwanchaikasem, Kaewta Rattanapisit, Waranyoo Phoolcharoen, Vudhiporn Limprasutr

**Affiliations:** 1 MSc. Program in Research for Enterprise, Faculty of Pharmaceutical Sciences, Chulalongkorn University, Bangkok 10330, Thailand; 2 Baiya Phytopharm Co., Ltd., Bangkok 10330, Thailand; 3 Center of Excellence in Plant-Produced Pharmaceuticals, Chulalongkorn University, Bangkok 10330, Thailand; 4 Department of Pharmacognosy and Pharmaceutical Botany, Faculty of Pharmaceutical Sciences, Chulalongkorn University, Bangkok 10330, Thailand; 5 Department of Pharmacology and Physiology, Faculty of Pharmaceutical Sciences, Chulalongkorn University, Bangkok 10330, Thailand; 6 Center of Excellence in Preclinical Toxicity and Efficacy Assessment of Medicines and Chemicals, Chulalongkorn University, Bangkok 10330, Thailand

**Keywords:** 1B18 monoclonal antibody, botulinum neurotoxin, ELISA, LC-MS/MS, mouse model, pharmacokinetics, plant-derived antibody

## Abstract

**Background:** Botulism, caused by *Clostridium botulinum* neurotoxins (BoNT/A and B), is a life-threatening disorder requiring rapid, effective therapeutic intervention. Plant-produced monoclonal antibodies offer a scalable alternative to conventional antibody manufacturing. This study evaluated the pharmacokinetic properties of 1B18 monoclonal antibody (mAb), transiently expressed in and extracted from tobacco (*Nicotiana benthamiana*), which binds both BoNT/A and BoNT/B with high affinity. **Methods:** Male ICR mice (*n* = 12/group) received intravenous or intramuscular doses of plant-produced 1B18 mAb (25–100 µg), with serum samples collected across pre-dose and 13 post-administration timepoints (totaling 14 timepoints). Antibody quantification was performed using ELISA and LC–MS/MS to assess consistency and reliability. **Results and Discussion:** Following intravenous administration, the mAb exhibited prolonged elimination with half-lives ranging from 40.4 to 56.0 h (at 25 and 50 µg doses). ELISA-derived absolute bioavailability was approximately 99% and 85% at the 50 and 100 µg intramuscular doses, demonstrating efficient systemic absorption comparable to intravenous administration. ELISA was used as the primary quantitative method for pharmacokinetic evaluation, whereas LC-MS/MS provided exploratory, sequence-specific support for the observed concentration–time profiles. Because many LC-MS/MS-derived concentrations were below the mathematically estimated LOQ, cross-platform comparisons were interpreted cautiously and were not considered evidence of analytical equivalence or interchangeability. **Conclusions:** These findings demonstrate that plant-derived 1B18 mAbs possess favorable pharmacokinetic profiles and high bioavailability, supporting their potential as long-acting therapeutic candidates for botulism treatment and advancing sustainable antibody production strategies.

## 1. Introduction

Human botulism is a severe neuroparalytic disease caused by BoNT, the most potent toxin known, produced by *Clostridium botulinum* [1,2,3]. Although seven serotypes of BoNT (A–G) have been identified [2,3,4], types A and B remain the primary drivers of foodborne botulism-related morbidity and mortality worldwide [3,5,6]. The global public health significance of these neurotoxins is underscored by the continued emergence of foodborne outbreaks in multiple countries, including Thailand [7], Vietnam [8], the United Kingdom [9], and the United States [10,11]. BoNT is a 150 kDa protein consisting of a 100 kDa heavy chain and a 50 kDa light chain. The receptor-binding domain of the heavy chain mediates the selective attachment of BoNT to presynaptic membranes at the neuromuscular junctions. Subsequently, the translocation domain of the heavy chain facilitates endocytosis of the BoNT-receptor complex into the neuron, where the heavy chain, a zinc-dependent endopeptidase, cleaves SNARE proteins essential for acetylcholine release [12,13]. This mechanism results in flaccid paralysis characterized by generalized muscle weakness, respiratory failure, and death [14], with a reported mortality rate of 5–10% among untreated cases [5]. The administration of anti-BoNT antibodies is the only specific and effective therapy to reduce mortality when administered early after diagnosis. These antibodies neutralized BoNT by binding to specific epitopes, as exemplified by the 1B18 mAbs, which targets the translocation domain of BoNT types A and B [14,15]. However, the global supply of highly specific mAbs remains constrained by manufacturing bottlenecks, prohibitive costs, and a reliance on mammalian-derived components such as equine serum, which carry the risk of hypersensitivity and severe allergic reactions [5,14,16,17,18,19].

Plant-based expression systems have emerged as a promising alternative for therapeutic antibody production. Plant-derived antibodies can mitigate the risks of serum-derived hypersensitivity while offering advantages such as reduced production costs, rapid scalability, and high yield [16,17,18,20]. Thus, the development of plant-produced anti-BoNT mAbs represents a feasible and sustainable strategy to improve therapeutic accessibility. Among BoNT serotypes, types A and B are the most prevalent and clinically significant in humans [4]. Accordingly, developing highly specific antibodies targeting BoNT/A and BoNT/B remains a major research priority. A previous study demonstrated that the plant-derived 1B18 mAbs exhibit strong binding affinity to both BoNT/A and BoNT/B [21].

To support further development toward clinical use, a thorough understanding of the PK properties of the 1B18 mAbs is essential. PK characterization provides critical information on absorption, distribution, metabolism, and elimination, which determines the antibody’s bioavailability, dosing regimen, and therapeutic window. For biologics such as mAbs, PK profiles can be influenced by factors including molecular size, route of administration, and potential immunogenicity. Evaluating the PK behavior of plant-produced 1B18 mAbs following IV and IM administration in ICR mice will provide insights into systemic exposure and duration of action, thereby supporting dose optimization and formulation design for subsequent preclinical and clinical studies. In addition, this study compares two analytical quantification methods, namely ELISA and LC–MS/MS, for measuring 1B18 mAb concentrations in mouse serum. ELISA is a widely used, sensitive, and cost-effective technique for quantifying antibodies [22], but it can be affected by cross-reactivity, matrix interference, and limited dynamic range [23]. LC–MS/MS, by contrast, offers superior specificity, wider dynamic range, and minimal matrix interference [22,24], though it requires specialized instrumentation and technical expertise. The comparative evaluation of ELISA and LC–MS/MS, together with PK profiling of plant-derived 1B18 mAbs, will inform appropriate bioanalytical method selection and provide foundational data for the nonclinical development of this novel plant-produced anti-botulinum therapeutic.

## 2. Results

### 2.1. PK Analysis by ELISA

#### 2.1.1. PK of 1B18 mAbs Following IV Administration

**Figure 1:**
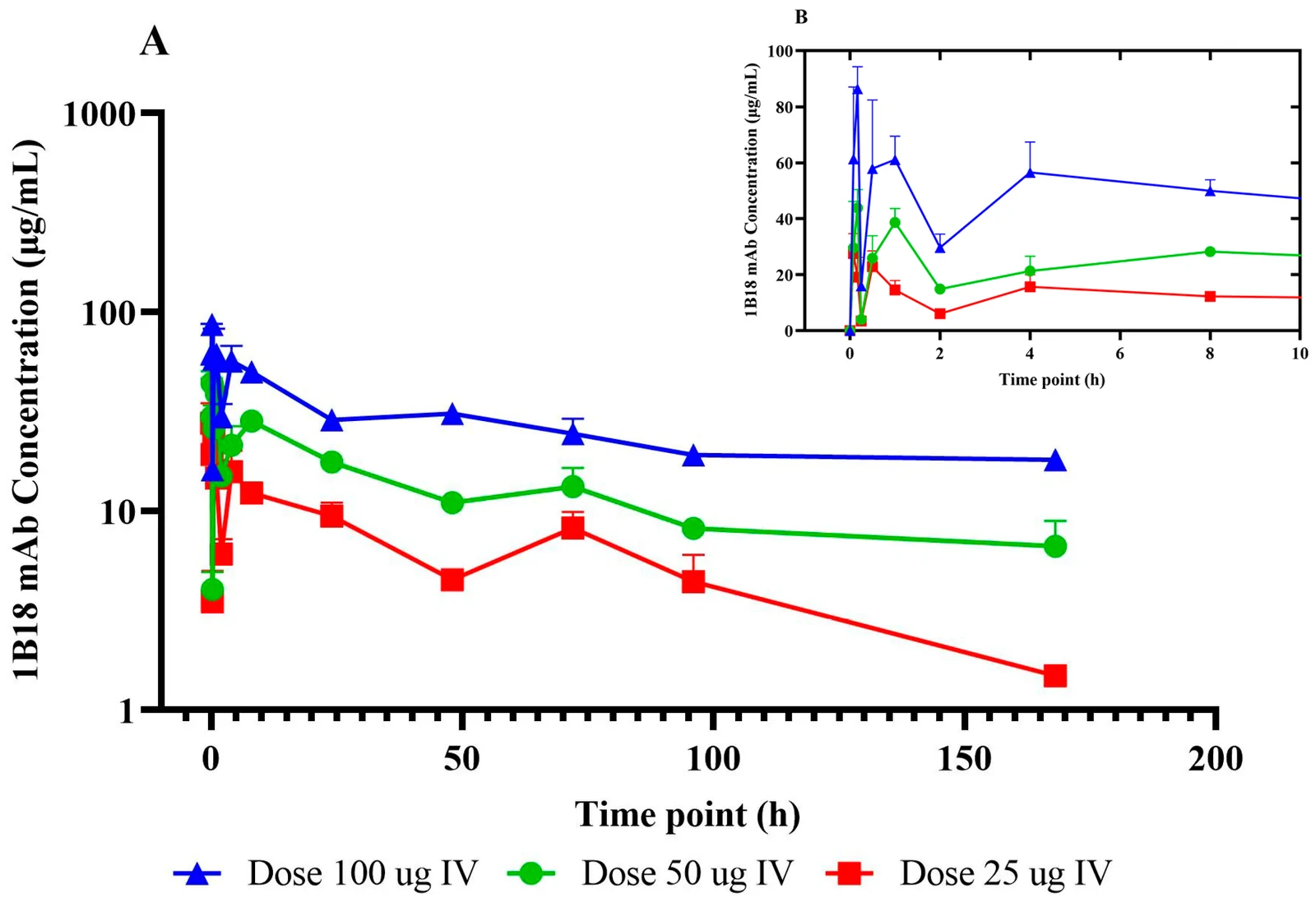
Serum concentration–time profiles of 1B18 mAb following IV administration at 25, 50, and 100 µg determined by ELISA analysis. Data are presented as mean ± SEM (*n* = 4 per timepoint for 0–24 h and *n* = 3 for 48–168 h). (**A**) Full concentration–time profiles from 0 to 168 h presented on a semi-logarithmic scale. (**B**) Expanded concentration–time profiles during the first 8 h presented on a linear scale.

The serum concentration–time profiles of plant-derived 1B18 mAbs following IV administration at doses of 25, 50, and 100 µg are illustrated in Figure 1. Serum concentrations were quantified by ELISA, and PK parameters were determined using a non-compartmental model applied to composite mean concentration–time profiles (Table 1), with regression diagnostics reported in Supplementary Table S1.

Following IV administration, C_max_ values of 1B18 mAbs were 27.60, 43.88, and 86.40 µg/mL at doses of 25, 50, and 100 µg, respectively, with T_max_ occurring between 0.08 and 0.17 h. The terminal elimination half-life (t_1/2_) was estimated to be 40.36 h and 56.03 h for the 25 and 50 µg groups, respectively. In contrast, the terminal elimination phase in the 100 µg group could not be reliably characterized (adjusted R^2^ = 0.732, %AUC_extra_ = 45.9%); therefore, t_1/2_ is reported as not estimable (NE) in Table 1 according to the predefined goodness-of-fit criteria (Supplementary Table S1).

As expected for intravenous bolus administration, peak exposure occurred rapidly (T_max_ ≤ 0.17 h). Furthermore, systemic exposure (C_max_) exhibited a consistent dose-dependent increase across cohorts. Similarly, the observed systemic exposure up to the last quantifiable timepoint (AUC_0−t_) increased with dose, yielding 961.46, 1915.24, and 4192.64 µg/mL·h for the 25, 50, and 100 µg cohorts, respectively (Table 1). While the terminal parameters for the highest dose cohort could not be mathematically resolved, quantifiable serum concentrations observed through 168 h confirmed persistent circulating antibody levels across all evaluated doses.

Overall, these findings demonstrate that the systemic exposure of plant-derived 1B18 mAbs following IV administration is dose-dependent, characterized by dose-dependent increases in C_max_ and AUC_0−t_ values with rapid systemic distribution (Table 1).

#### 2.1.2. PK of 1B18 mAbs Following IM Administration

The PK profile of plant-derived 1B18 mAbs following IM administration was characterized using ELISA. The PK parameters obtained from non-compartmental analysis (NCA) and one-compartment model analysis (1CA) applied to composite mean profiles are summarized in Table 1, with regression diagnostics reported in Supplementary Table S1.

After IM administration at doses of 25, 50, and 100 µg/mouse, the observed T_max_ ranged from 48 to 72 h, with corresponding C_max_ values of 6.56, 18.88, and 35.65 µg/mL, respectively. Using NCA, the terminal elimination t_1/2_ was reliably estimated at 57.42 h for the 50 µg group (Adjusted R^2^ = 0.963, %AUC_extra_ = 19.7%). In contrast, terminal phases for the 25 µg (Adjusted R^2^ = 0.095) and 100 µg (Adjusted R^2^ = 0.667, %AUC_extra_ = 28.6%) cohorts failed to satisfy predefined goodness-of-fit criteria and were designated as NE (Table 1, Supplementary Table S1). Observed systemic exposure up to 168 h (AUC_0−t_) increased across cohorts, yielding 622.22, 1894.44, and 3563.62 µg/mL·h for the 25, 50, and 100 µg groups, respectively. These exposures resulted in absolute bioavailability (F) of 64.72%, 98.91%, and 84.99% for the 25, 50, and 100 µg groups, respectively (Table 1).

**Figure 2:**
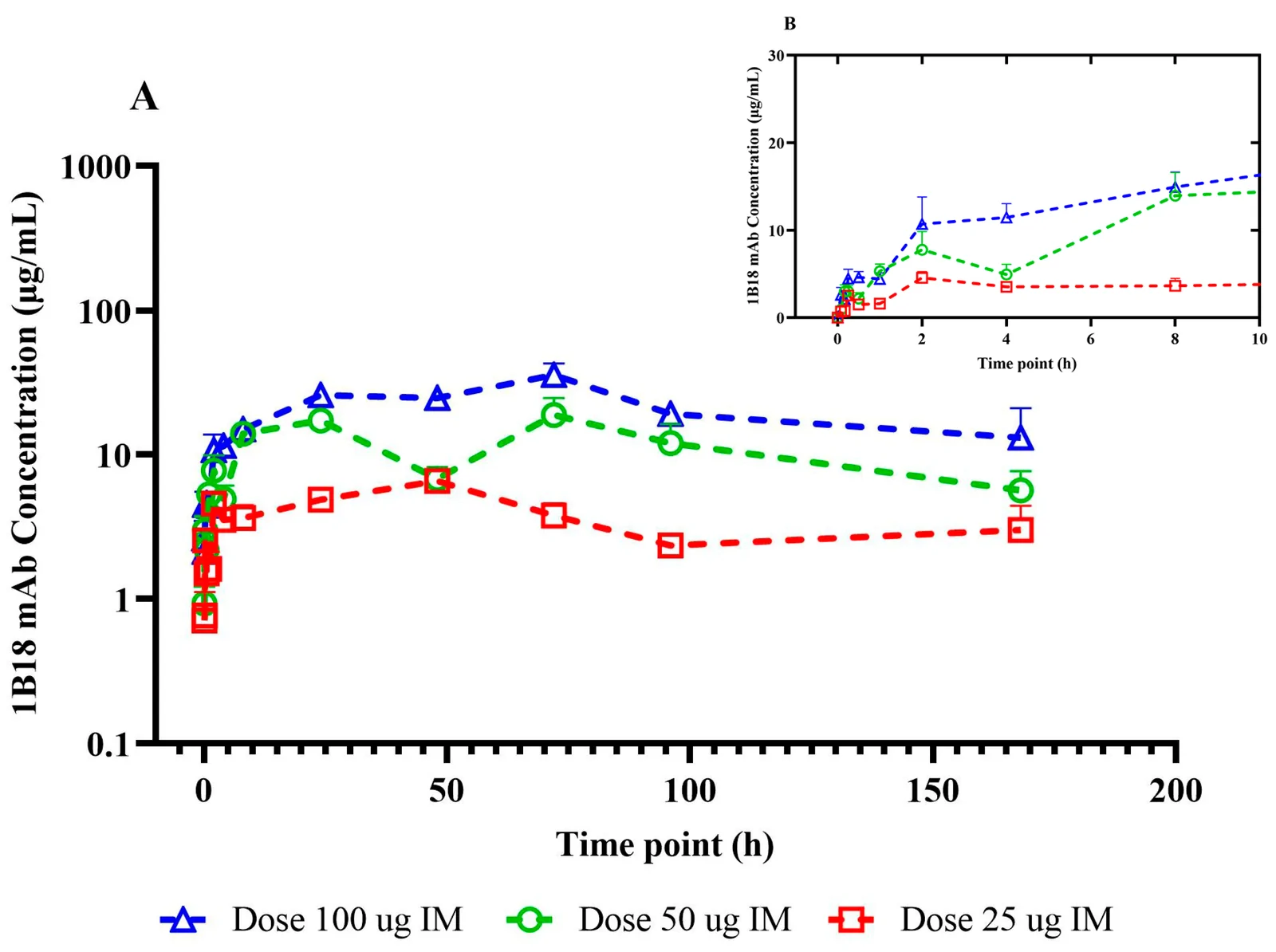
Serum concentration–time profiles of 1B18 mAb following intramuscular administration at 25, 50, and 100 µg determined by ELISA analysis. Data are presented as mean ± SEM (*n* = 4 per timepoint for 0–24 h and *n* = 3 for 48–168 h). (**A**) Full concentration–time profiles from 0 to 168 h presented on a semi-logarithmic scale. (**B**) Expanded concentration–time profiles during the first 8 h presented on a linear scale.

Compartmental modeling using an extravascular one-compartment model described the initial absorption and distribution profiles across cohorts (Figure 2). The estimated absorption half-lives (t_1/2 abs_) were 0.78, 3.52, and 8.64 h for the 25, 50, and 100 µg doses, respectively, corresponding to the model-predicted T_max_ values of 6.63, 19.35, and 35.32 h, and C_max_ values of 4.56, 15.53, and 29.56 µg/mL (Table 1). The model-predicted AUC values were 629.68, 1926.43, and 3711.19 µg/mL·h, respectively. However, due to prolonged IM absorption and sustained post-peak exposure, the model exhibited structural parameter non-identifiability (k_10_ → 0); consequently, the t_1/2_ values from the 1CA were designated as NE across all doses in accordance with Table 1. Overall, these evaluations demonstrated dose-dependent increases in systemic exposure (C_max_ and AUC) accompanied by characteristic absorption delays typical of extravascularly administered large protein therapeutics.

#### 2.1.3. Comparison of PK Profiles Following IV and IM Administration Analyzed by ELISA

**Figure 3:**
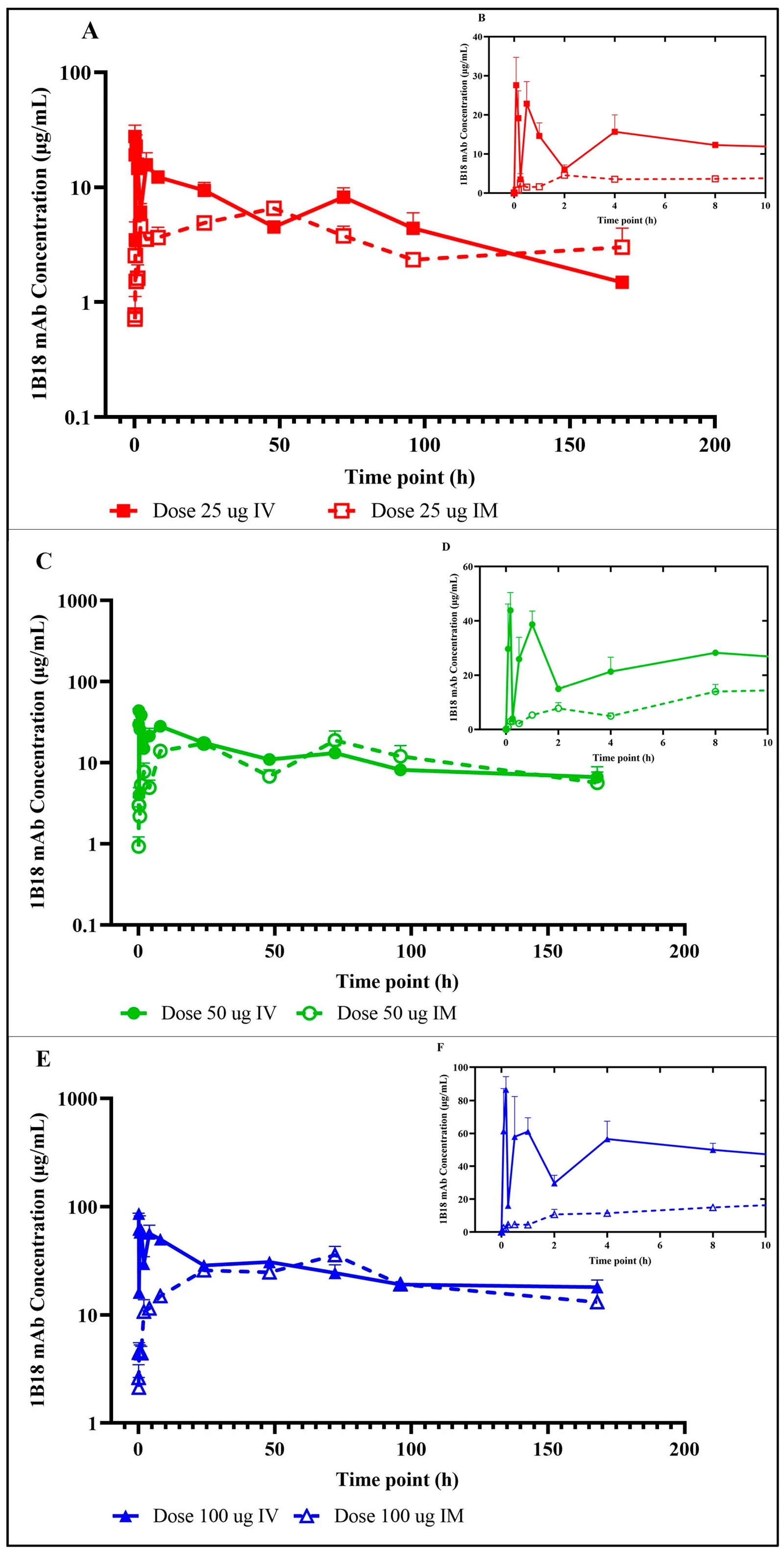
Comparative pharmacokinetic analysis of serum concentration–time profiles of 1B18 mAb following IV and IM administration. Profiles are shown for (**A**,**B**) 25 µg, (**C**,**D**) 50 µg, and (**E**,**F**) 100 µg cohorts determined by ELISA analysis. Data are presented as mean ± SEM (*n* = 4 per timepoint for 0–24 h and *n* = 3 for 48–168 h). (**A**,**C**,**E**) Full concentration–time profiles from 0 to 168 h presented on a semi-logarithmic scale. (**B**,**D**,**F**) Expanded concentration–time profiles during the first 8 h presented on a linear scale.

The PK parameters of plant-derived 1B18 mAbs against BoNT were compared between IV and IM administration at doses of 25, 50, and 100 µg/mouse, based on ELISA (Figure 3). The comparison focused on key PK parameters, including t_1/2_, T_max_, C_max_, and AUC derived from composite mean profiles (Table 1). For the 50 µg cohort, where terminal phases met predefined goodness-of-fit criteria, the apparent t_1/2_ were highly comparable between routes (56.03 h for IV vs. 57.42 h for IM, as shown in Table 1). In contrast, terminal half-lives for the other cohorts (25 µg IM, 100 µg IV, and 100 µg IM) did not satisfy the terminal regression criteria and were designated as NE (Table 1, Supplementary Table S1).

IM administration exhibited a characteristically delayed absorption phase compared with IV administration, with observed T_max_ occurring between 48 and 72 h across doses, compared to the prompt peak exposure observed for IV bolus injection (0.08–0.17 h; Table 1). Consequently, C_max_ values were consistently lower following IM administration, reflecting gradual absorption from the injection site into the systemic circulation. Similarly, observed systemic exposure (AUC_0−t_) was lower following IM than IV administration at the 25 µg (622.22 vs. 961.46 µg/mL·h) and 100 µg (3563.62 vs. 4192.64 µg/mL·h) dose levels. In contrast, systemic exposure was comparable between the two routes at the 50 µg dose (1894.44 vs. 1915.24 µg/mL·h for IM and IV administration, respectively; Table 1).

Bioavailability (F), calculated as the AUC_0−t_ ratio of IM to IV, was 64.72%, 98.91%, and 84.99% for the 25, 50, and 100 µg doses, respectively, indicating high systemic availability of 1B18 mAbs following IM administration, particularly at intermediate and high doses.

### 2.2. PK Analysis by LC-MS/MS

#### 2.2.1. PK of 1B18 mAbs Following IV Administration

**Figure 4:**
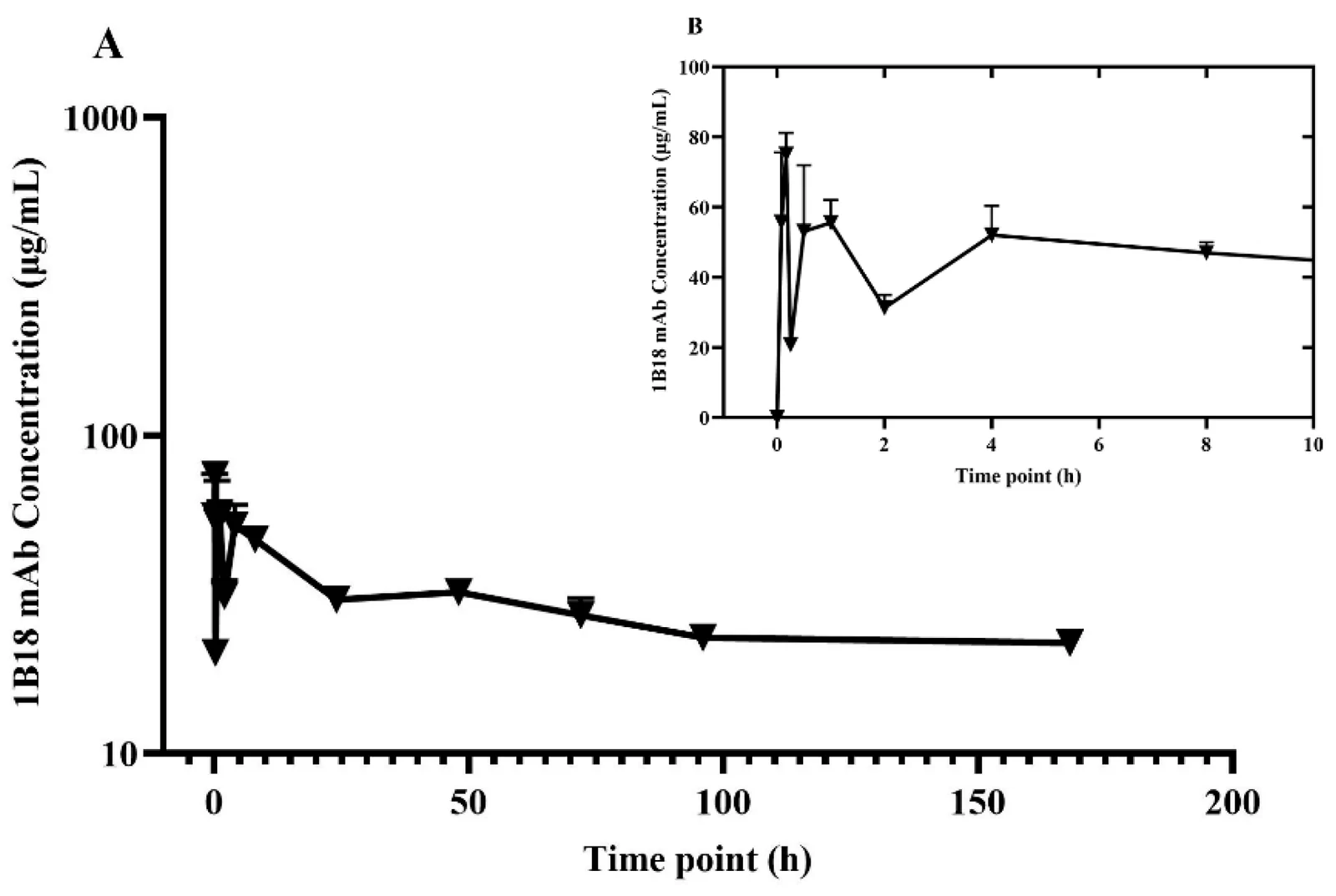
Serum concentration–time profiles of 1B18 mAb following IV administration at 100 µg determined by LC-MS/MS analysis. Data are presented as mean ± SEM (*n* = 4 per timepoint for 0–24 h and *n* = 3 for 48–168 h). (**A**) Full concentration–time profiles from 0 to 168 h presented on a semi-logarithmic scale. (**B**) Expanded concentration–time profiles during the first 8 h presented on a linear scale.

The PK characteristics of plant-derived 1B18 mAbs targeting BoNT were evaluated following IV administration at a dose of 100 µg/mouse. Serum concentrations of 1B18 mAbs were quantified using LC-MS/MS, and the resulting concentration–time profiles are shown in Figure 4. The NCA PK parameters are summarized in Table 1.

Following IV administration, the highest observed LC-MS/MS-derived concentration was 75.06 µg/mL at 0.17 h (Table 1). Systemic exposure up to the last quantifiable sampling point (AUC_0−t_) was 4692.16 µg/mL·h. However, the apparent terminal elimination phase failed to satisfy the predefined goodness-of-fit criteria due to poor regression linearity and excessive extrapolation ($R_{\mathrm{adj}}^{2}<0.80$, %AUC_extra_ > 20%); consequently, the t_1/2_ was designated as NE (Table 1). Measurable chromatographic signals through 168 h provided sequence-specific evidence of the persistence of the selected 1B18-derived surrogate peptide in serum. These findings do not independently confirm the molecular integrity or biological activity of the circulating antibody.

#### 2.2.2. PK of 1B18 mAbs Following IM Administration

**Figure 5:**
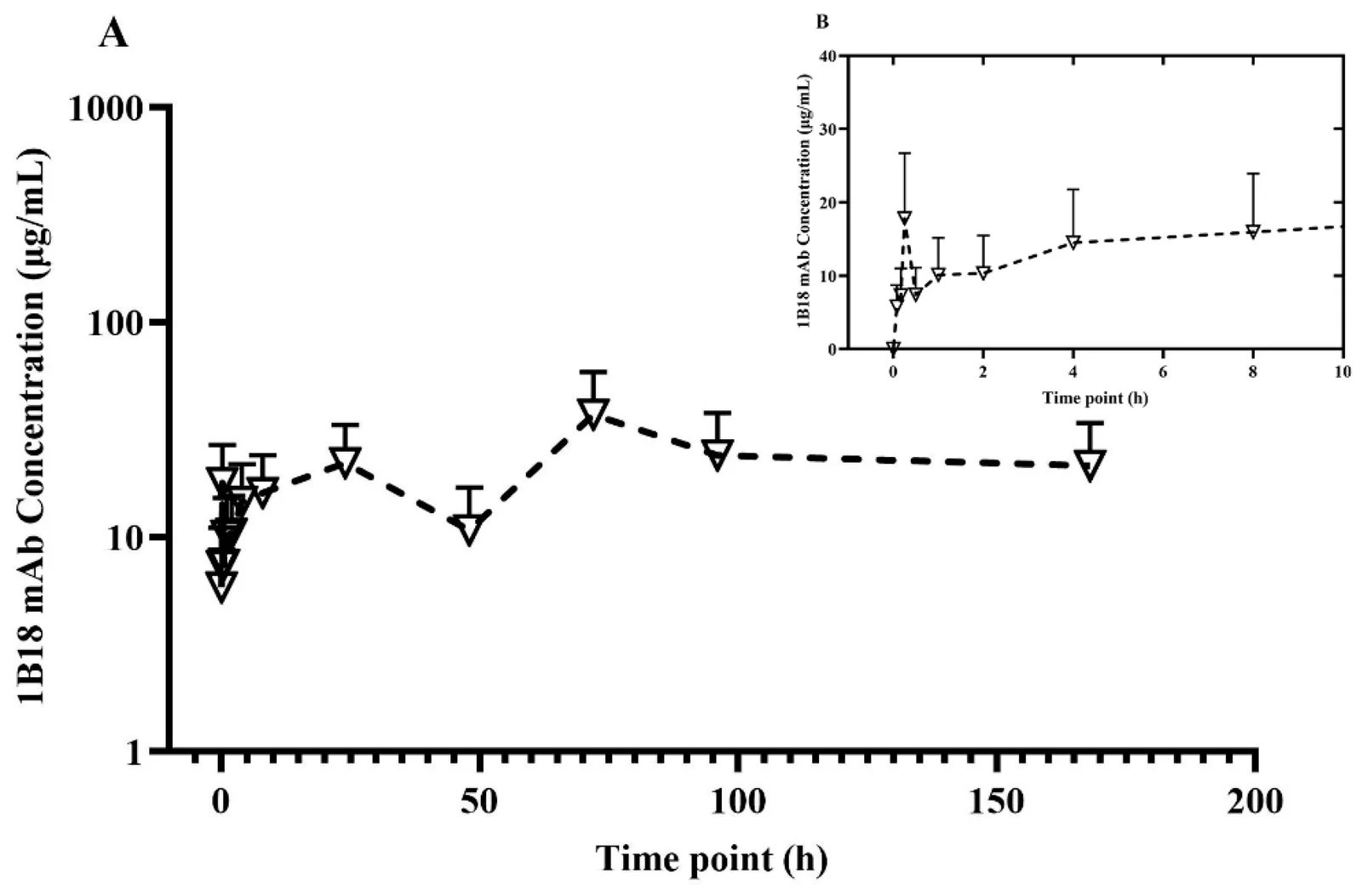
Serum concentration–time profiles of 1B18 mAb following IM administration at 100 µg determined by LC-MS/MS analysis. Data are presented as mean ± SEM (*n* = 4 per timepoint for 0–24 h and *n* = 3 for 48–168 h). (**A**) Full concentration–time profiles from 0 to 168 h presented on a semi-logarithmic scale. (**B**) Expanded concentration–time profiles during the first 8 h presented on a linear scale.

The serum concentration–time profile of plant-derived 1B18 mAbs following IM administration at a dose of 100 µg/mouse was analyzed using LC-MS/MS (Figure 5). The pharmacokinetic parameters derived from NCA and an extravascular 1CA are summarized in Table 1.

Following IM administration, the highest observed LC-MS/MS-derived concentration was 37.01 µg/mL at 48 h (Table 1). However, the terminal elimination phase did not meet predefined regression criteria ($R_{\mathrm{adj}}^{2}\geq 0.80$ and %AUC_extra_ < 20%; Supplementary Table S1); consequently, the t_1/2_ was designated as NE rather than reported numerically (Table 1, Supplementary Table S1). AUC_0−t_ was 3735.05 µg/mL was designated as an exploratory estimate (Table 1).

To further evaluate absorption kinetics, an extravascular 1CA was applied. The t_1/2 abs_ was 1.25 h, with a predicted T_max_ of 8.28 h and a corresponding C_max_ of 20.27 µg/mL. However, the 1CA exhibited parameter non-identifiability (k_10_ approached zero) due to the absence of a distinct log-linear terminal slope; therefore, the model-derived t_1/2_ and compartmental exposure metrics were designated as NE (Table 1), consistent with the persistent plateau observed post-C_max_ (Figure 5).

The serum concentration–time profile of plant-derived 1B18 mAbs following IM administration at a dose of 100 µg/mouse was analyzed using LC–MS/MS (Figure 5). The pharmacokinetic parameters derived from NCA and an extravascular 1CA are summarized in Table 1.

#### 2.2.3. Comparison of PK Profiles Between IV and IM Administration Analyzed by LC-MS/MS

**Figure 6:**
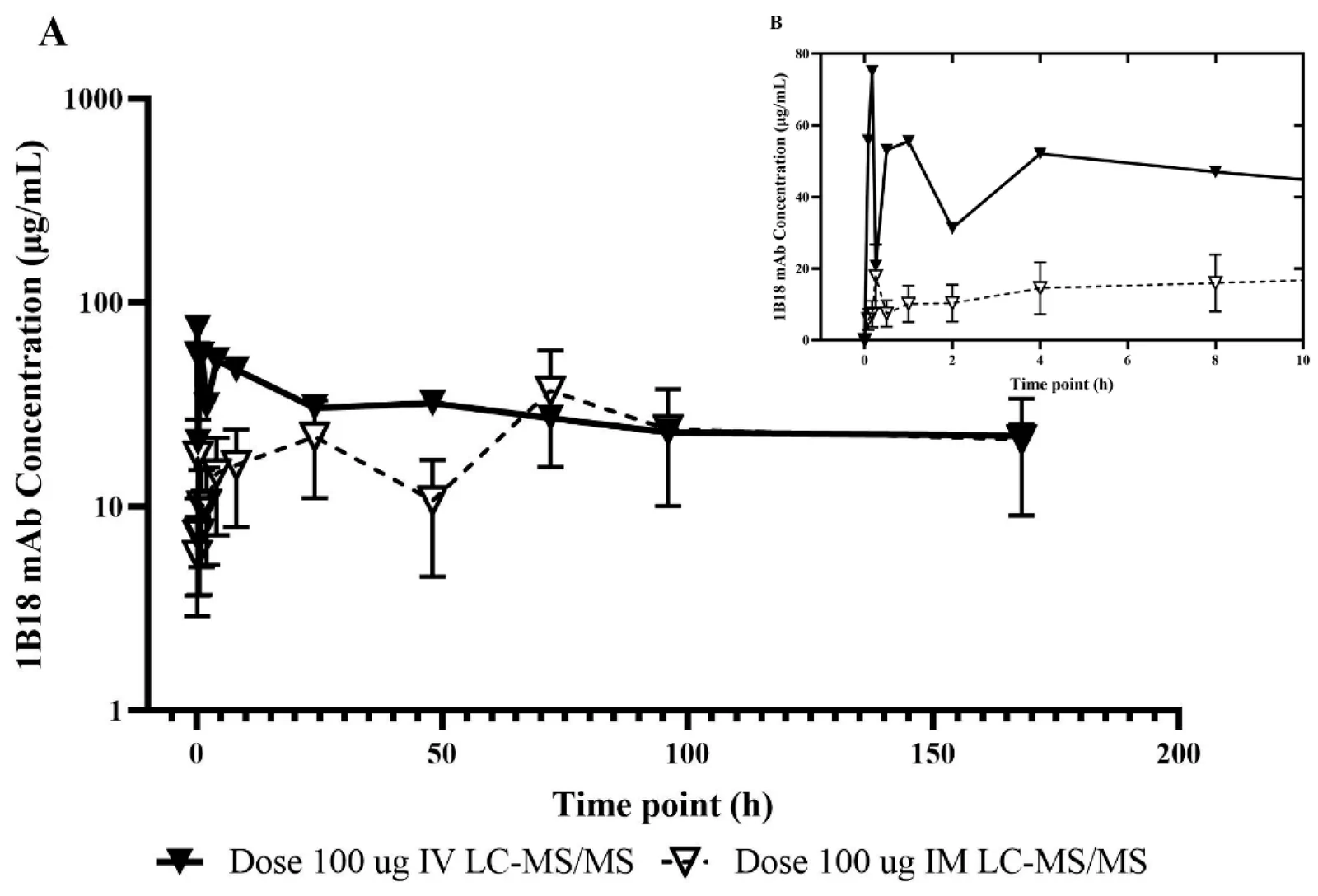
Comparative pharmacokinetic analysis of serum concentration–time profiles of 1B18 mAb following IV and IM administration at 100 µg, determined by LC-MS/MS analysis. Data are presented as mean ± SEM (*n* = 4 per timepoint for 0–24 h and *n* = 3 for 48–168 h). (**A**) Full concentration–time profiles from 0 to 168 h presented on a semi-logarithmic scale. (**B**) Expanded concentration–time profiles during the first 8 h presented on a linear scale.

The PK profiles of plant-derived 1B18 mAbs following IV and IM administration at a dose of 100 µg/mouse were compared using LC–MS/MS (Figure 6). The PK parameters derived from NCA and 1CA applied to composite mean profiles are summarized in Table 1, with diagnostic regression parameters detailed in Supplementary Table S1.

Following IV administration, peak exposure occurred immediately with a T_max_ of 0.17 h and a C_max_ of 75.06 µg/mL (Table 1). In contrast, IM administration exhibited a characteristically prolonged absorption phase, reaching a T_max_ of 48 h with a lower C_max_ of 37.01 µg/mL (Table 1). The apparent t_1/2_ for both routes failed to satisfy the predefined goodness-of-fit criteria ($R_{\mathrm{adj}}^{2}\geq 0.80$ and %AUC_extra_ < 20%) and were strictly designated as NE (Table 1). The LC-MS/MS profiles showed an early peak following IV administration and a delayed peak following IM administration. However, because many LC-MS/MS-derived concentrations were below the mathematically estimated LOQ, the associated AUC and bioavailability estimates were considered exploratory and were not used to support definitive quantitative conclusions regarding systemic exposure or absorption efficiency.

To evaluate extravascular absorption kinetics, an extravascular one-compartment model was applied to the IM profile. The estimated t_1/2 abs_ was 1.25 h, with a model-predicted T_max_ of 8.28 h and a corresponding C_max_ of 20.27 µg/mL (Table 1). However, the one-compartment model failed to reliably resolve the elimination rate constant (k_10_ approached zero) due to the persistent post-peak curve plateau; consequently, model-derived terminal parameters (t_1/2_ and compartmental AUC) exhibited structural non-identifiability and were designated as NE in Table 1, confirming that compartmental terminal estimates for this profile should not be used for definitive clearance interpretations.

#### 2.2.4. Comparison of PK Data Analyzed by ELISA and LC-MS/MS

**Figure 7:**
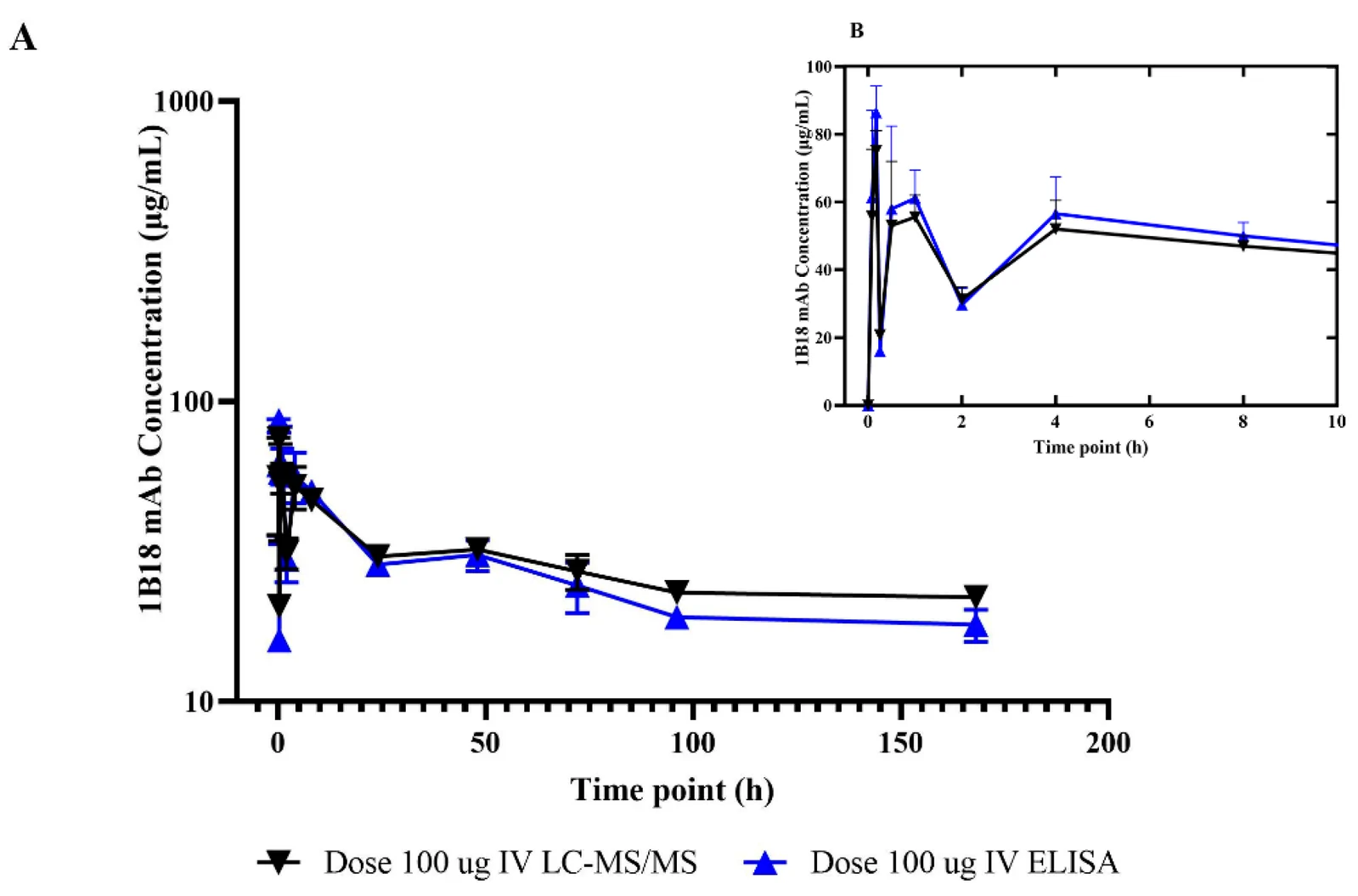
Comparison of ELISA and LC-MS/MS analysis for serum concentration–time profiles of 1B18 mAb following IV administration at 100 µg. Data are presented as mean ± SEM (*n* = 4 per timepoint for 0–24 h and *n* = 3 for 48–168 h). (**A**) Full concentration–time profiles from 0 to 168 h presented on a semi-logarithmic scale. (**B**) Expanded concentration–time profiles during the first 8 h presented on a linear scale.

**Figure 8:**
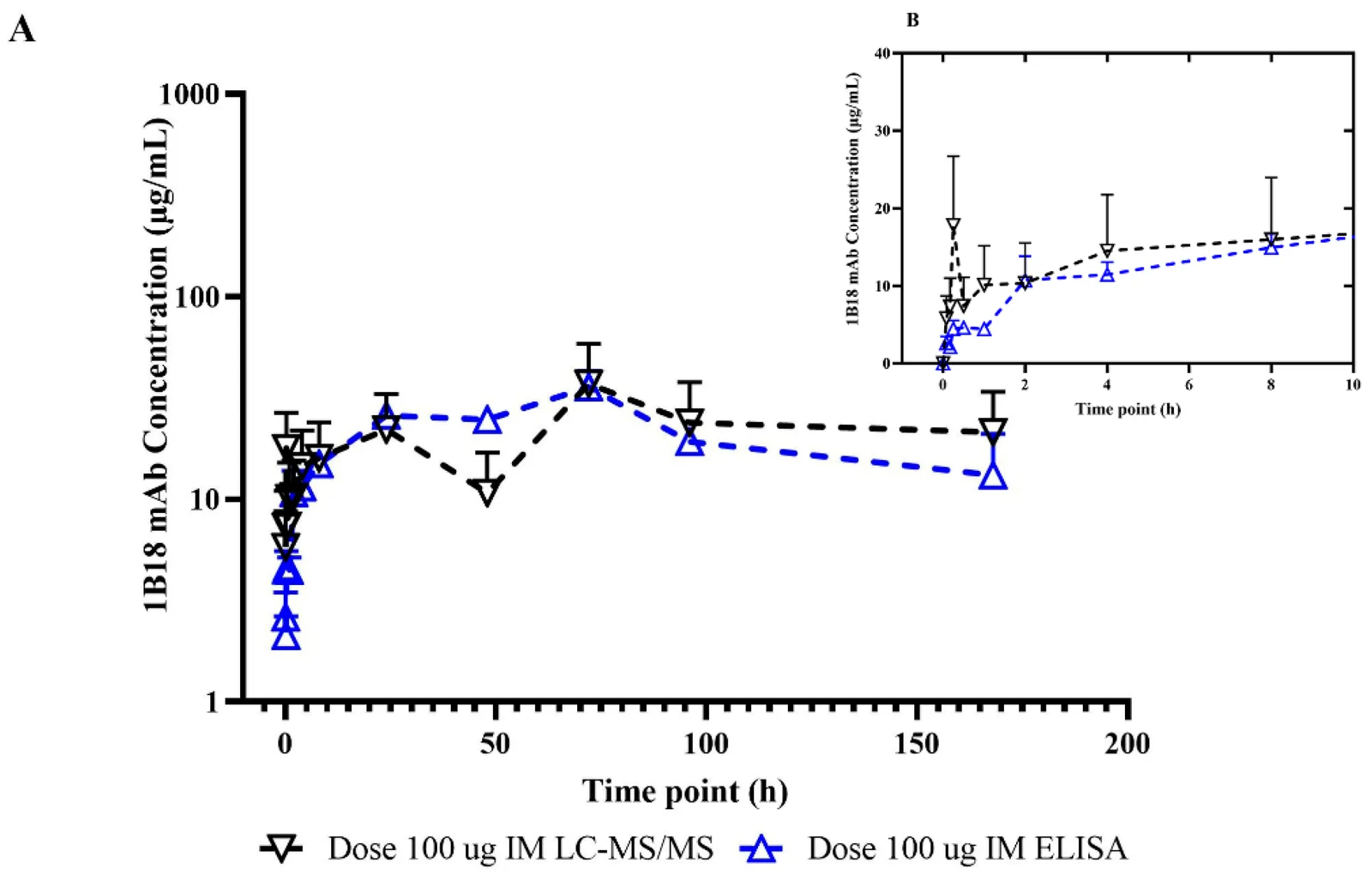
Comparison of ELISA and LC-MS/MS analysis for serum concentration–time profiles of 1B18 mAb following IM administration at 100 µg. Data are presented as mean ± SEM (*n* = 4 per timepoint for 0–24 h and *n* = 3 for 48–168 h). (**A**) Full concentration–time profiles from 0 to 168 h presented on a semi-logarithmic scale. (**B**) Expanded concentration–time profiles during the first 8 h presented on a linear scale.

The concentration–time profiles of the 1B18 mAbs obtained from ELISA and LC-MS/MS analyses following IV and IM administration at a dose of 100 µg/mouse are presented in Figure 7 and Figure 8, respectively, with corresponding composite NCA pharmacokinetic parameters summarized in Table 1. Both analytical platforms consistently captured route-dependent exposure characteristics, demonstrating rapid systemic entry with high peak concentrations following IV delivery (T_max_ = 0.17 h), contrasting with prolonged absorption (T_max_ = 48 − 72 h) and attenuated peak exposure following IM injection (Table 1). For both analytical methods, t_1/2_ for the 100 µg cohorts failed predefined goodness-of-fit criteria and were designated as NE (Table 1), directing cross-platform evaluations toward observed concentrations and systemic exposure metrics (C_max_ and AUC_0−t_).

**Figure 9:**
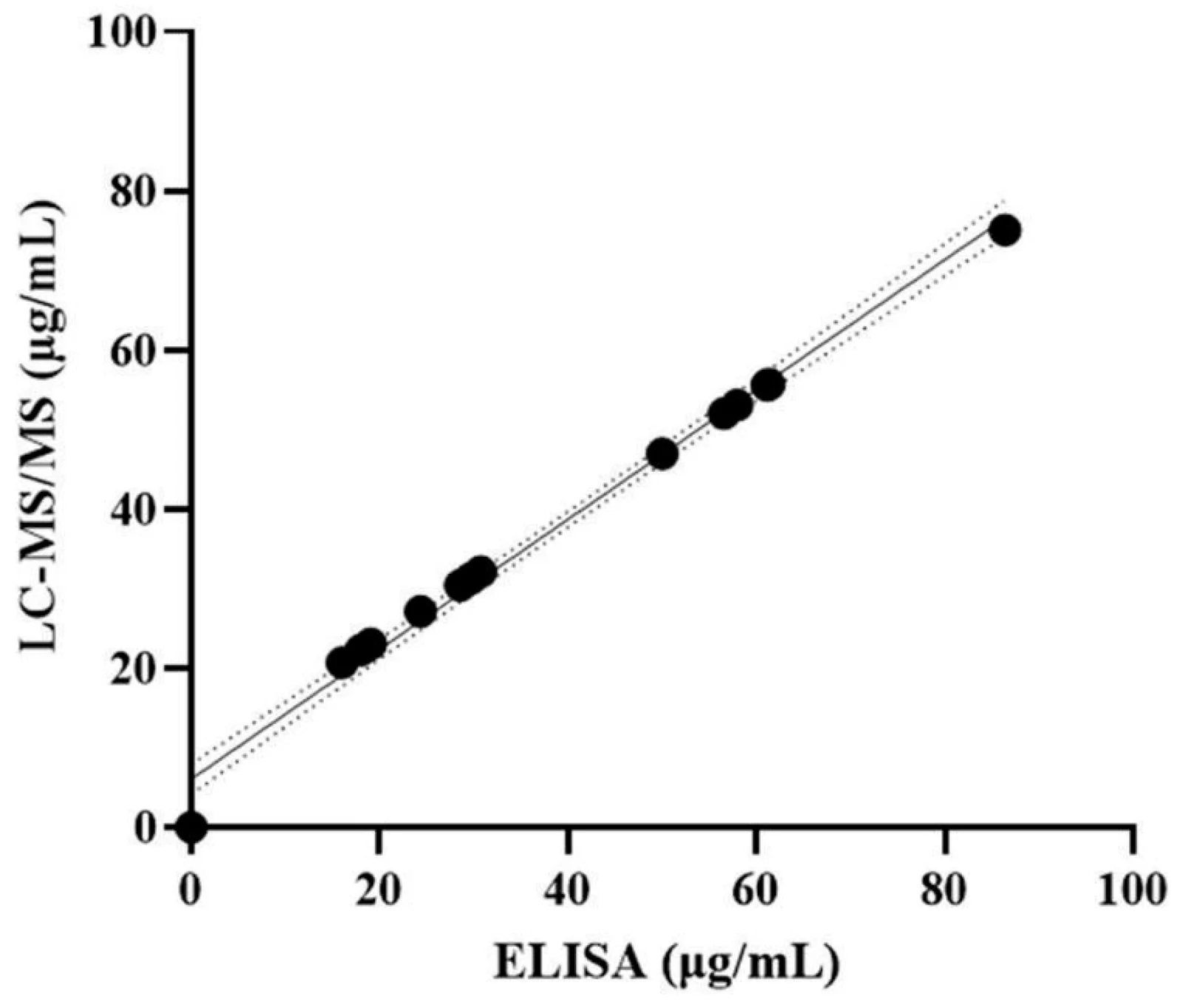
Comparison of 1B18 mAbs concentration–time data obtained from LC-MS/MS and ELISA after IV administration (100 µg/mouse). The solid line represents the line of best fit, and dashed lines represent 95% confidence intervals (95% CI = 0.74 − 0.87).

**Figure 10:**
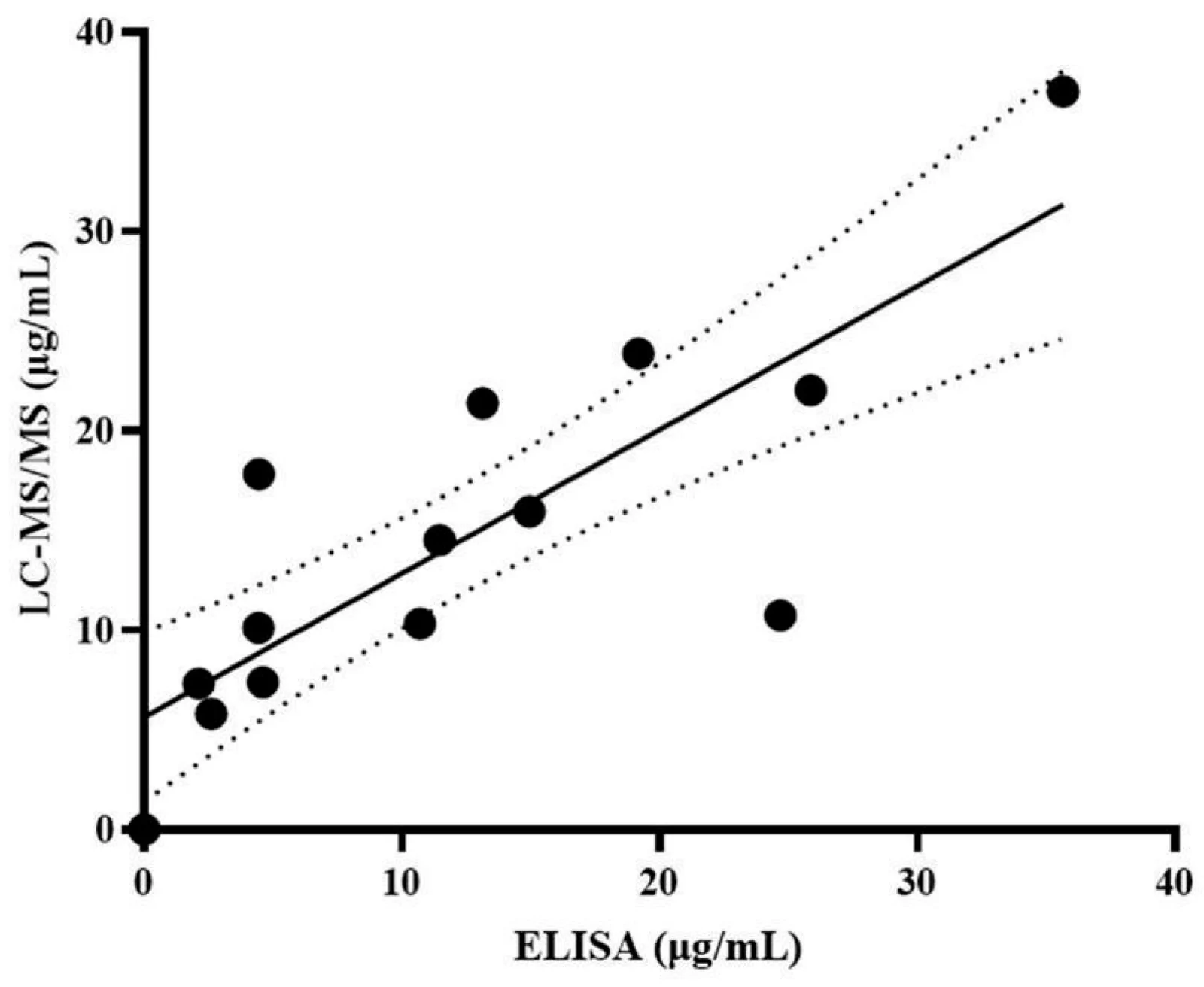
Comparison of 1B18 mAbs concentration–time data obtained from LC-MS/MS and ELISA after IM administration (100 µg/mouse). The solid line represents the line of best fit, and dashed lines represent 95% confidence intervals (95% CI = 0.40–1.04).

Cross-platform agreement between LC-MS/MS and ELISA was formally evaluated using linear regression, ANCOVA, and Bland–Altman analysis (Table 2, Supplementary Table S2, Figure 9 and Figure 10). Linear regression analysis revealed a strong correlation between platforms across the quantified concentration ranges, with r^2^ = 0.99 for IV and r^2^ = 0.66 for IM delivery (Table 2). ANCOVA detected no statistically significant differences in the fitted slopes or elevations; however, the absence of statistical significance was not interpreted as evidence of analytical equivalence (Supplementary Table S2). In addition, Bland–Altman analysis demonstrated acceptable concordance, with minimal mean biases of 1.031 µg/mL (95% LoA: −8.406 to 10.47 µg/mL) for IV and −2.174 µg/mL (95% LoA: −14.34 to 9.993 µg/mL) for IM administration, alongside mean ratios of 1.04 and 1.69, respectively (Table 2).

Collectively, these findings indicate acceptable cross-platform concordance between LC–MS/MS and ELISA for tracking circulating 1B18 mAb concentrations. While ELISA remains the primary platform providing superior sensitivity across the full dynamic range, the sequence-specific LC–MS/MS assay serves as a robust orthogonal tool, providing supportive evidence of the temporal presence of the selected 1B18-derived surrogate peptide.

## 3. Discussion

This study characterized the preclinical PK profile of plant-derived 1B18 mAb against BoNT types A and B [1,2,3,5,6]. Rapid neurotoxin neutralization is clinically critical to prevent paralysis and reduce mortality in botulism [25,26]. Plant-based biomanufacturing offers rapid scalability and superior biosafety compared to mammalian production [20,27,28,29,30], crucial for emergency biodefense and global clinical access. The major findings demonstrate that 1B18 mAb exhibits dose-dependent systemic exposure (C_max_ and AUC_0−t_) following IV administration, whereas IM delivery shows characteristic delayed extravascular absorption while maintaining robust systemic exposure and high absolute bioavailability (~80–99% at intermediate and high doses; Table 1). These PK behaviors, evaluated through both complementary bioanalytical methods, provide essential baseline evidence supporting the potential of plant-based therapeutics.

Following IV administration, dose-dependent increases in C_max_ and AUC_0−t_ were observed with consistent, rapid T_max_ (0.08–0.17 h), reflecting immediate systemic availability (Table 1). Robust t_1/2_ were estimable for the lower dose cohorts (40.36 to 56.03 h for 25 and 50 µg doses). However, the 100 µg IV profile exhibited an indistinct terminal elimination phase with excessive extrapolation (>20%), failing the predefined goodness-of-fit criteria and requiring designation as NE (Table 1, Supplementary Table S1). Consequently, potential dose-dependent alterations in terminal clearance could not be conclusively resolved within the 168 h sampling window. These exposure characteristics nevertheless demonstrate rapid systemic bioavailability, providing a solid framework for future studies exploring 1B18 mAb in acute therapeutic interventions.

IM administration yielded dose-dependent extravascular absorption profiles characterized by delayed T_max_ (48–72 h) and less-than-dose-dependent increases in C_max_, consistent with rate-limiting lymphatic uptake and local retention typical of large protein therapeutics [31,32,33]. Despite lower peak concentrations compared to IV administration, robust AUC_0−t_ was achieved, conferring favorable absolute bioavailability (~65–99% across cohorts, reaching ~85–99% at intermediate and high doses; Table 1). An extravascular 1CA was applied to describe IM kinetics. However, sustained depot release and post-peak curve flattening masked the terminal elimination phase, resulting in mathematical parameter non-identifiability (k_10_ approached zero); consequently, these model-derived terminal parameters were designated as NE (Table 1). This underscores that prolonged extravascular absorption can compromise terminal elimination modeling, warranting strict exclusion of these unidentifiable parameters from quantitative clearance evaluations.

A key translational hurdle is the altered glycosylation profile of plant-derived mAbs, which may reduce human FcRn binding affinity [34,35,36]. This could potentially accelerate clearance and shorten t_1/2_ in humans compared to mice. These findings offer important implications for future anti-botulinum therapeutic strategies. The robust systemic exposure achieved via IM injection suggests a potential alternative administration route where IV access is unavailable. However, considering the delayed T_max_ observed for IM delivery in this study, further preclinical evaluations in active toxin-challenge models are required before establishing its suitability for emergency or field-based applications. To successfully advance this candidate, future studies must incorporate detailed PK/PD modeling and carefully design dosing schedules to compensate for potentially altered FcRn interactions.

From an analytical perspective, ELISA served as the primary quantitative platform because of its substantially greater sensitivity across the concentration range observed in this study. LC-MS/MS provided complementary, sequence-specific detection of a selected 1B18-derived surrogate peptide. However, the LC-MS/MS method was evaluated using only one QC concentration, and many study-sample concentrations were below the mathematically estimated LOQ of 78.72 µg/mL. Therefore, the LC-MS/MS-derived concentration estimates, the resulting exposure parameters (such as AUC_0−t_), and cross-platform analyses were regarded as exploratory. The observed similarities in temporal profiles should not be interpreted as evidence of quantitative equivalence or analytical interchangeability. Further validation using multiple QC levels, appropriate weighting, and an isotope-labelled internal standard would be required before LC-MS/MS could be used as a definitive quantitative method for PK assessment.

Several limitations of this study should be explicitly acknowledged. First, the PK evaluations were conducted exclusively in healthy male ICR mice and utilized a sparse sampling design, which necessitated the use of composite concentration–time profiles rather than individual pharmacokinetic analysis. Second, the 168 h observation period was relatively short to fully capture the terminal clearance phase for several dose cohorts; consequently, these profiles failed our predefined terminal goodness-of-fit criteria ($R_{\mathrm{adj}}^{2}\geq 0.80$ and %AUC_extra_ < 20%) and were strictly designated as NE (Table 1 and Supplementary Table S1), precluding definitive terminal clearance interpretations for those groups. Third, this study did not include an active botulinum toxin challenge model; thus, the actual in vivo therapeutic protection and neutralization kinetics remain to be verified. Finally, a direct experimental comparison with a conventionally produced mammalian-cell-derived 1B18 antibody was not included in this study, which limits direct conclusions regarding their comparative pharmacokinetic equivalence.

Overall, the PK findings demonstrate that the plant-derived 1B18 mAbs persist in circulation and offer excellent bioavailability in mice, highlighting the potential of plant-based systems as a scalable and efficient platform for producing anti-BoNT treatments. To advance this candidate toward Phase I clinical trials, future research should focus on glycoengineering the plant host to produce human-like sugar structures. This modification could potentially improve the antibody’s interaction with FcRn receptors and extend its half-life in the body. Finally, to ensure it is safe and effective for human use, further studies must evaluate the antibody in larger animal models during active toxin exposure, alongside thorough safety, efficacy, and molecular integrity assessments.

## 4. Materials and Methods

### 4.1. Production of 1B18 Monoclonal Antibodies

The 1B18 mAb is a plant-derived human IgG1 antibody developed against BoNT serotypes A and B (Baiya Phytopharm Co., Ltd., Bangkok, Thailand) using a transient expression system in *Nicotiana benthamiana* plants. Recombinant *Agrobacterium tumefaciens* strains harboring the light-chain (50 kDa) and heavy-chain (100 kDa) constructs were cultured separately and subsequently co-infiltrated into *N. benthamiana* via vacuum-assisted agroinfiltration. Four days after infiltration, the leaves were harvested, and crude protein was extracted using phosphate-buffered saline (PBS; 137 mM NaCl, 2.7 mM KCl, 8.1 mM Na_2_HPO_4_, 1.5 mM KH_2_PO_4_, pH 7.4; Sigma-Aldrich, St. Louis, MO, USA).

Purification of 1B18 mAbs was performed using Protein A affinity chromatography. The column was equilibrated and washed with 1 × PBS, and bound antibodies were eluted with 0.1 M sodium citrate buffer (9.38 mM C_6_H_9_ Na_3_O_9_, 90.62 mM C_6_H_8_O_7_, pH 3.0), followed by immediate neutralization with 1.5 M Tris-HCl (pH 8.8). The antibody concentration was determined by sandwich ELISA. Briefly, 96-well plates were coated with goat polyclonal anti-human IgG Fc-specific antibody (ab97221, Abcam, Cambridge, UK) and blocked appropriately. The antigen-binding specificity of purified 1B18 mAbs was verified by ELISA using modified BoNT/A1 (mBoNT/A1) and chimeric BoNT/B1 (ciBoNT/B1) proteins [21] as coating antigens. Bound antibodies were detected using horseradish peroxidase (HRP)-conjugated anti-human kappa antibodies (Southern Biotech, Birmingham, AL, USA). The colorimetric reaction was developed using 3,3′,5,5′-tetramethylbenzidine (TMB; Promega^®^, Madison, WI, USA), stopped with 1 M H_2_SO_4_, and absorbance was measured at 450 nm using a microplate reader (Hercuvan Lab Systems, London, UK).

### 4.2. Animals

All animal procedures were conducted in accordance with protocol no. 24-33-004, approved by the Institutional Animal Care and Use Committee (IACUC) of the Faculty of Pharmaceutical Sciences, Chulalongkorn University, following the Guide for the Care and Use of Laboratory Animals (8th edition). A total of 72 male ICR mice (*Mus musculus*), five to eight weeks of age, were obtained from Nomura Siam International Co., Ltd. (Bangkok, Thailand). All animals were confirmed to be free from common murine pathogens, including Dermatophytes, *Citrobacter rodentium*, *Pasteurella pneumotropica*, *Corynebacterium kutscheri*, *Pseudomonas aeruginosa*, *Salmonella* spp., *Staphylococcus aureus*, *Mycoplasma pulmonis*, *Clostridium piliforme*, Mouse hepatitis virus, Sendai virus, Ectromelia virus, *Giardia muris*, *Spironucleus muris*, *Syphacia* spp., *Aspiculuris tetraptera*, and ectoparasites.

Mice weighing 36–46 g were quarantined for 7 days before randomization and group-housed in an AAALAC-accredited facility. Animals received standard rodent chow and sterile drinking water ad libitum. Environmental conditions were maintained at 22 ± 2 °C, 40–60% relative humidity, and a 12:12 h light–dark cycle.

Animals were monitored daily for general health, behavior, and signs of pain or distress. Immediately following mAb administration, clinical monitoring was performed every 2 h for the first 6 h, every 4 h up to 24 h post-dose, and twice daily thereafter throughout the 168 h study period. Humane endpoints were established based on clinical signs, including severe lethargy, inability to access food or water, rapid body weight loss exceeding 20%, or marked respiratory distress, which would mandate immediate humane euthanasia via CO_2_ asphyxiation in accordance with the AVMA Guidelines for the Euthanasia of Animals (2020). No animals exhibited adverse clinical signs or met the criteria for humane endpoints during the study.

### 4.3. Experimental Design

A comparative PK study was conducted in male ICR mice (*n* = 72) to characterize the PK profile of plant-derived 1B18 mAbs following IV and IM administration. To reduce pain and stress during injections, mice were gently secured using appropriately sized, well-ventilated restrainers. Mice were randomly assigned to six groups (12 mice/group) representing three dose levels (25, 50, and 100 µg) for each route. Each group was divided into three subgroups (*n* ≥ 3 animals each) for blood sampling at designated timepoints. The sample size was calculated to achieve 95% statistical power at α = 0.05. Randomization was performed using an online random number generator (https://www.graphpad.com/quickcalcs/randomize1/), accessed on 15 May 2025.

### 4.4. PK Studies

**Figure 11:**
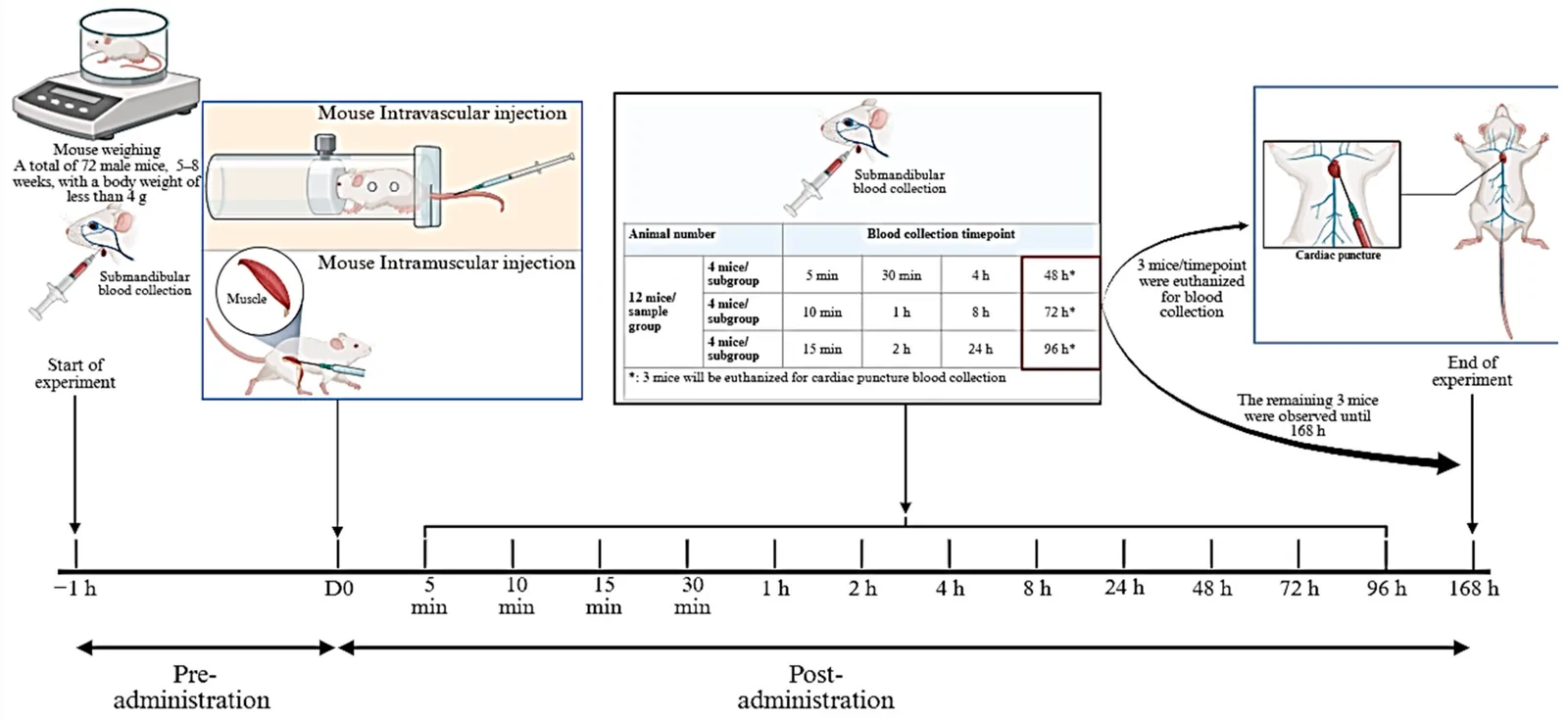
PK study design.

Each mouse received a single dose of plant-derived 1B18 mAbs via IV or IM injection using a 0.5 mL syringe with a 29G × ½ inch needle (NIPRO, Bangkok, Thailand). Blood samples were collected from the submandibular vein at pre-dose and 13 post-administration timepoints: 0.08, 0.17, 0.25, 0.5, 1, 2, 4, 8, 24, 48, 72, 96, and 168 h, as shown in Figure 11 (Created with BioRender.com, licensed under CC BY 4.0). At 48, 72, 96, and 168 h, mice of each group were euthanized via CO_2_ asphyxiation in accordance with the AVMA Guidelines for the Euthanasia of Animals (2020) for cardiac blood collection. Specifically, samples were obtained from 4 animals per timepoint from pre-dose up to 24 h (*n* = 4) and from 3 animals per timepoint at 48, 72, and 96 h (*n* = 3). For the final 168 h timepoint, one mouse from each of the three subgroups was sampled (total *n* = 3).

Serum samples were isolated by centrifugation (3000 rpm, 15 min) and stored at −20 °C until analysis. Quantification of serum 1B18 mAbs concentrations was performed independently using ELISA and LC–MS/MS. All analysts were blinded to treatment groups.

### 4.5. Quantity Testing of Anti-Botulinum mAbs

#### 4.5.1. ELISA

Quantification of serum anti-BoNT mAbs was performed using an indirect ELISA method. Microtiter plates were coated overnight at 4 °C with goat anti-human IgG-HRP antibody (SouthernBiotech, Birmingham, AL, USA) at a concentration of 2 µg/mL. Plates were washed three times with 1X PBST (1X PBS containing 0.05% Tween 20; Sigma-Aldrich, St. Louis, MO, USA) and blocked with 5% (*w*/*v*) skim milk (BD Diagnostic, Franklin Lakes, NJ, USA) in PBS buffer (137 mM NaCl, 2.7 mM KCl, 8.1 mM Na_2_HPO_4_, and 1.5 mM KH_2_PO_4_, pH 7.4) for 2 h at 37 °C.

Following blocking, diluted mouse serum samples and protein standards were added and incubated for 2 h at 37 °C. After washing, plates were incubated with peroxidase-conjugated goat anti-human kappa antibody (SouthernBiotech, Birmingham, AL, USA) for 1 h at 37 °C. Color development was achieved with 3,3′,5,5′-tetramethylbenzidine (TMB One Solution, Promega, Madison, WI, USA) for 15 min at room temperature and stopped by adding 1 M sulfuric acid (Ajax Finechem, Seven Hills, Australia).

Absorbance was measured at 450 nm using an Ensight Multimode Reader (PerkinElmer, Shelton, CT, USA). To account for potential non-specific binding and matrix interference, assay controls, including blank mouse serum (matrix blank) and 1X PBS (reagent negative control), were incorporated into each plate, and background absorbance was subtracted accordingly. A standard calibration curve was generated using a four-parameter logistic (4PL) regression model to determine the antibody concentrations in serum samples. The calibration curve demonstrated an excellent fit (R^2^ = 0.9997). The lower limit of quantification (LLOQ) and upper limit of quantification (ULOQ) for the assay were established at 0.0016 µg/mL and 0.4 µg/mL, respectively. Within this quantifiable range, the assay exhibited high quantitative performance with precision (%CV) ranging from 0.92% to 6.80% and accuracy ranging from 91.0% to 112.1%.

#### 4.5.2. Liquid Chromatography-Mass Spectrometry (LC-MS/MS)

Quantification of 1B18 mAbs in mouse serum was performed using LC-MS/MS following direct enzymatic digestion without prior immunoassay enrichment [37]. Calibration standards across the range of 5 to 200 µg/mL (5, 10, 20, 35, 50, 85, 100, 135, 150, 185 and 200 µg/mL) were prepared by spiking serial dilutions of 1B18 reference protein into blank mouse serum. For sample preparation, 20 µL of mouse serum sample (or calibration standard) was mixed with 10 µL of internal standard (IS, Wuhan RBD-Fc at a working concentration of 20 µg/mL) and 10 µL of ultrapure water (Wako Pure Chemical Industries, Ltd., Osaka, Japan). (IS, Wuhan RBD-Fc). Protein precipitation was induced by adding 160 µL of 100% methanol (RCI Labscan, Bangkok, Thailand). A recombinant Wuhan-RBD–Fc fusion protein was employed as an internal standard (IS). This construct shares structural similarity with the analyte by possessing an immunoglobulin Fc domain, thereby paralleling 1B18 mAb during sample processing, while exhibiting a distinct sequence with no cross-homology to 1B18 or endogenous mouse serum proteins. Its in-house availability also provided an accessible, cost-effective surrogate to track recovery and enzymatic processing variability across runs.

Samples were gently inverted and centrifuged at 4000× *g* for 5 min. The resulting pellet was resuspended in 40 µL of 50 mM ammonium bicarbonate buffer (Sigma-Aldrich, St. Louis, MO, USA). Reduction and alkylation were performed by sequential addition of 5 µL of 100 mM dithiothreitol (Glentham LIFE SCIENCES, Corsham, UK) at 65 °C for 30 min and 2.5 µL of iodoacetamide (Glentham LIFE SCIENCES, Corsham, UK) at room temperature in the dark for 20 min. Proteolytic digestion was initiated by adding 0.25 µg of trypsin (Trypsin Gold, Thermo Scientific, Waltham, MA, USA) and incubating at 37 °C for 4 h. The reaction was terminated with 5 µL of 10% formic acid, and samples were centrifuged at 14,000× *g* for 10 min. A 5 µL aliquot of the supernatant was subjected to LC-MS/MS analysis. Chromatographic separation was achieved using a Zorbax Eclipse Plus C18 column (2.1 × 50 mm, 1.8 µm; Agilent, Santa Clara, CA, USA) maintained at 40 °C, coupled to a 6495D triple quadrupole mass spectrometer (Agilent, Santa Clara, CA, USA). The mobile phase consisted of 0.1% formic acid (FA) in water (A) and 0.1% FA in acetonitrile (B), with the following gradient: 0–0.5 min, 0% B; 0.5–1 min, 0–25% B; 1–5 min, 25–35% B; 5–8 min, 35–100% B; and 8–10 min, 0% B. The surrogate peptide for 1B18 mAb light chain was LLIFAASSLQSAVPSR. The multiple reaction monitoring (MRM) transition was 830.5 to 359.2 *m*/*z* with a collision energy (CE) of 17 eV as a quantifier and 641.3 to 301.2 with a CE of 16 eV as a qualifier. The surrogate peptide for the IS protein was QIAPGGTGK, and its MRM transition was 450.3 to 587.3 *m*/*z* with a CE of 15 eV. MS settings were as follows: gas temperature of 200 °C, drying gas flow of 14 L/min, nebulizer at 20 psi, sheath gas temperature of 250 °C, and sheath gas flow of 11 L/min. Capillary and nozzle voltages were set at 3000 V and 1500 V, respectively. The analysis was performed in positive ionization mode. Retention times for 1B18 and IS peptides were 2.1 and 1.9 min, respectively. The relative peak area of the 1B18 peptide detected from the samples was compared against that of the linear standard curve to quantify the 1B18 mAb level in the serum.

The LC-MS/MS method was developed as a fit-for-purpose exploratory method for sequence-specific detection of the 1B18 surrogate peptide in mouse serum. A linear calibration model was evaluated over the nominal concentration range of 5–200 µg/mL. The mathematical LOQ, calculated as 10 times the residual standard deviation divided by the calibration slope, was 78.72 µg/mL, whereas the corresponding estimated LOD was in the range of 20–30 µg/mL. These values were mathematical estimates and were not considered experimentally validated LLOQ and LOD values. Method performance was assessed using a single QC concentration of 75 µg/mL, with intra-day accuracy of 95.29%, overall inter-day accuracy of 90.73%, and the %CV of multiple injections of QC samples was lower than 4.20%. Because full validation across multiple QC levels was not performed and many study-sample concentrations were below the mathematical LOQ, LC-MS/MS-derived concentrations in this range were regarded as semi-quantitative and exploratory. Accordingly, the LC-MS/MS data were used as supportive, sequence-specific evidence and not as the primary basis for quantitative PK or terminal-phase interpretation.

### 4.6. Data Analysis for PK Profile

Serum concentration–time data were expressed as mean ± standard error of the mean (SEM) and analyzed to determine the PK parameters of plant-derived 1B18 mAbs. PK evaluation was performed using both non-compartmental analysis (for IV and IM administration) and one-compartment model analysis (for IM administration) with PK-Solver Software (version 2.0) [38].

Because full individual concentration–time profiles could not be obtained from single animals, the mean plasma concentrations at each timepoint were used to construct a single composite concentration–time profile for each dose group (*n* ≥ 3). Consequently, all pharmacokinetic parameters were derived from these composite profiles.

Key PK parameters, including clearance (CL), apparent terminal elimination half-life (t_1_/_2_), area under the concentration–time curve (AUC), maximum serum concentration (C_max_), time to reach maximum concentration (T_max_), elimination rate constant (Ke), and apparent volume of distribution (Vd), were determined. C_max_ (µg/mL) represents the highest observed serum concentration, and T_max_ (h) indicates the time at which this concentration occurs. The elimination rate constant (Ke, h^−1^) describes the slope of the terminal phase of the plasma concentration–time curve and is related to both CL and Vd. Clearance reflects the rate of systemic elimination and was calculated as CL = Dose/AUC. The Vd represents the theoretical volume in which the antibody is distributed and was calculated as Vd = Dose/C_0_, where C_0_ is the extrapolated initial concentration. The terminal elimination t_1_/_2_ was calculated as t_1_/_2_ = 0.693/Ke, representing the time required for the serum concentration to decrease by half during the elimination phase.

The terminal elimination rate constant (λz) was estimated via unweighted log-linear regression of the terminal phase of the serum concentration–time profile. The selection of data points for λz determination was governed by the following criteria:

Inclusion of at least three consecutive timepoints in the terminal log-linear phase, excluding C_max_;

An adjusted coefficient of determination Adjusted R^2^ ≥ 0.80; and

A percentage of extrapolated AUC_extra_ < 20%. Profiles failing to satisfy these goodness-of-fit criteria were designated as NE and excluded from terminal parameter comparisons.

To characterize the IM kinetics, an extravascular one-compartment model was employed. This model was selected to account for the prolonged absorption phase typical of large molecules administered intramuscularly. However, due to the potential for flip-flop kinetics where prolonged absorption masks the elimination phase, terminal parameters derived from this model were interpreted with caution. Bioavailability was estimated as $F=\frac{\mathrm{AU}C_{\mathrm{IM}}\times \mathrm{Dos}e_{\mathrm{IV}}}{\mathrm{AU}C_{\mathrm{IV}}\times \mathrm{Dos}e_{\mathrm{IM}}}\times 100$, indicating the fraction of the administered dose reaching systemic circulation following IM injection. These analyses were performed to characterize the absorption, distribution, and elimination kinetics of the plant-derived 1B18 mAbs in mice and to compare systemic exposure following IV and IM administration.

For NCA pharmacokinetic analysis of LC–MS/MS data, serum concentrations below the theoretical LOQ (78.72 µg/mL) but within the instrumentally discernible standard calibration range (down to the lowest calibration level of 5 µg/mL) were retained at their measured values to trace the empirical profile of circulating mAb. These values were treated as semi-quantitative, and all resulting exposure parameters (e.g., AUC_0−t_) were strictly designated as exploratory estimates.

### 4.7. Statistical Analysis

All statistical analyses were conducted using GraphPad Prism software (version 10.6.1; GraphPad Software, San Diego, CA, USA). Mean serum concentration–time profiles were subsequently constructed for each dosing group. Pharmacokinetic parameters were calculated from these composite mean concentration–time curves using PKSolver add-in for Microsoft Excel (version 2.0; China Pharmaceutical University, Nanjing, China). Because derived parameters represent single-point estimates from composite profiles, standard deviations and formal hypothesis testing (e.g., ANOVA) were not applied to secondary PK parameters (AUC, C_max,_ t_1/2_); parameter evaluations were conducted via descriptive comparison and regression analyses.

For the comparison between analytical methods (ELISA vs. LC–MS/MS) using the same biological samples, a paired Student’s *t*-test or Wilcoxon matched-pairs signed-rank test was applied depending on data distribution. Additionally, a Bland–Altman analysis was performed to evaluate the agreement between the two analytical methods.

A *p*-value of less than 0.05 (*p* < 0.05) was considered statistically significant, and exact *p*-values are reported where applicable.

## 5. Conclusions

This study characterized the pharmacokinetic profile of plant-derived 1B18 mAb following IV and IM administration in mice. ELISA-based analysis demonstrated dose-dependent systemic exposure, rapid peak concentrations following IV administration, and delayed absorption with high observed bioavailability following IM administration. LC–MS/MS provided exploratory, sequence-specific support for the observed temporal profiles but was not considered quantitatively interchangeable with ELISA because many measurements were below the mathematically estimated LOQ. These findings provide preliminary PK information to guide longer-duration studies, further bioanalytical validation, efficacy evaluation in toxin-challenge models, and subsequent development of plant-produced 1B18 mAb.

## Figures and Tables

**Table 1:** Pharmacokinetic parameters of plant-derived 1B18 mAb following intravenous and intramuscular administration in mice, determined using ELISA and exploratory LC-MS/MS analyses.

1B18 mAbs Analysis	PK Model	Administration Route	Dose	t_1/2 abs_^a^	t_max_^b^	C_max_^c^	t_1/2_^d^	AUC ^e^	Dose-Normalized		F
			µg/Mouse	h	h	μg/mL	h	µg/mL·h	$\frac{C_{\max}}{\mathrm{Dose}}$	$\frac{\mathrm{AUC}}{\mathrm{Dose}}$	(%)
ELISA	NCA	IV	100	-	0.17	86.40	NE	4192.64	0.86	41.93	-
			50	-	0.17	43.88	56.03	1915.24	0.88	38.30	-
			25	-	0.08	27.60	40.36	961.46	1.10	38.46	-
		IM	100	-	72.00	35.65	NE	3563.62	0.36	35.64	84.99
			50	-	72.00	18.88	57.42	1894.44	0.38	37.89	98.91
			25	-	48.00	6.56	NE	622.22	0.26	24.89	64.72
	1CA	IM	100	8.64	35.32	29.56	NE	3711.19	0.30	37.11	-
			50	3.52	19.35	15.53	NE	1926.43	0.31	38.53	-
			25	0.78	6.63	4.56	NE	629.68	0.18	25.19	-
LC-MS/MS	NCA	IV	100	-	0.17	75.06	NE	4692.16	0.75	46.92	-
		IM	100	-	48.00	37.01	NE	3735.05	0.37	37.35	80
	1CA	IM	100	1.25	8.28	20.27	NE	NE	0.20	NE	-

a: absorption half-life; b: time to reach maximum concentration of mAbs; c: maximum concentration; d: terminal elimination half-life; e: area under the curve; F: bioavailability; NE: not estimable; parameter failed predefined terminal-phase goodness-of-fit criteria (R^2^adj ≥ 0.80, %AUC_extra_ < 20%) or exhibited model parameter non-identifiability. LC–MS/MS-derived exposure parameters (e.g., C_max_, AUC_0−t_) are considered exploratory estimates, as several lower-range concentrations resided below the mathematical LOQ threshold.

**Table 2:** Summary of regression analysis comparing LC-MS/MS and ELISA quantification of 1B18 mAb following IV and IM administration in mice.

Group	Regression Equation	SEM.EST ^*^	Mean Bias (±SEM) ^‡^	Mean Ratio ^†^ (±SEM) ^§^	Range of Ratio
IV	Y = 0.8158X + 6.075 (r^2^ = 0.99)	2.06	1.03 ± 1.29	1.04 ± 0.03	0.87–1.29
IM	Y = 0.7206X + 5.643 (r^2^ = 0.66)	5.67	−2.17 ± 0.66	1.69 ± 0.29	0.44–3.99

Y: concentration of mAbs from LC-MS/MS; X: concentration of mAbs from ELISA; *: standard error of the estimate (standard error of the regression slope); ‡: mean and standard error (SEM) of the difference (LC-MS/MS-ELISA) between methods; †: ratio between LC-MS/MS and the reference method (i.e., LC − MS/MS ÷ ELISA; §: standard error of the ratios.

## Data Availability

The data presented in this study are available on request from the corresponding author due to the confidential under a non-disclosure agreement with Baiya Phytopharm Co., Ltd., and cannot be shared publicly.

## Supplementary Materials

The following supporting information can be downloaded at: https://www.mdpi.com/article/10.3390/ph19101597/s1, Table S1: Goodness-of-Fit and Diagnostic Parameters for Terminal Phase (λ_z) Estimation across Analytical Platforms and Dosing Cohorts; Table S2: Linear Regression Parameters and ANCOVA Comparison across Administration Routes and Analytical Platforms; Table S3: LC-MS/MS Method Validation.

## Abbreviations

%AUC_extra_	Percentage of Extrapolated Area Under the Curve
λz	Terminal Elimination Rate Constant
1CA	One-Compartment Analysis/One-Compartment Model
4PL	Four-Parameter Logistic
AAALAC	Association for Assessment and Accreditation of Laboratory Animal Care International
ANCOVA	Analysis of Covariance
ANOVA	Analysis of Variance
AUC	Area Under the Concentration–Time Curve
AUC_0−t_	Area Under the Concentration–Time Curve from Time Zero to the Last Quantifiable Timepoint
AVMA	American Veterinary Medical Association
BoNT	Botulinum Neurotoxin
C_0_	Back-Extrapolated Initial Concentration at Time Zero
C_max_	Maximum Observed Serum Concentration
CE	Collision Energy
CI	Confidence Interval
CL	Systemic Clearance
CV	Coefficient of Variation
ELISA	Enzyme-Linked Immunosorbent Assay
F	Absolute Bioavailability
FA	Formic Acid
FcRn	Neonatal Fc Receptor
HRP	Horseradish Peroxidase
IACUC	Institutional Animal Care and Use Committee
IgG/IgG1	Immunoglobulin G/Immunoglobulin G Subclass 1
IM	Intramuscular/Intramuscularly
IS	Internal Standard
IV	Intravenous/Intravenously
k_10_	Elimination Rate Constant from the Central Compartment
LC–MS/MS	Liquid Chromatography–Tandem Mass Spectrometry
LLOQ	Lower Limit of Quantification
LOD	Limit of Detection
LoA	Limits of Agreement
LOQ	Limit of Quantification
*m*/*z*	Mass-to-Charge Ratio
mAb	Monoclonal Antibody
MRM	Multiple Reaction Monitoring
NCA	Non-Compartmental Analysis
NE	Not Estimable
PK	Pharmacokinetics
PK/PD	Pharmacokinetic/Pharmacodynamic
QC	Quality Control
$R_{\mathrm{adj}}^{2}$	Adjusted Coefficient of Determination
RBD	Receptor-Binding Domain
SD	Standard Deviation
SEM	Standard Error of the Mean
t_1/2_	Terminal Elimination Half-Life
t_1/2 abs_	Absorption Half-Life
T_max_	Time to Reach Maximum Observed Concentration
ULOQ	Upper Limit of Quantification
V_d_	Apparent Volume of Distribution
