# Supplementary material for: Plant-Derived Monoclonal Antibody Against Botulinum Neurotoxin: Pharmacokinetic Evaluation in a Mouse Model
Source: Pharmaceuticals (Basel). 2026 Oct 9;19(10):1597. doi: 10.3390/ph19101597 (PMC13708563; doi:10.3390/ph19101597)
Supplement: Supplementary file 1 [file pharmaceuticals-19-01597-s001.zip › Pharmaceuticals-4553574_Supplementary_PK Tables S1&S2.pdf]

Supplementary Plant-Derived Monoclonal Antibody Against Botulinum Neurotoxin: Pharmacokinetic Evaluation in a Mouse Model

Table S1. Goodness-of-Fit and Diagnostic Parameters for Terminal Phase (λ_z) Estimation across Analytical Platforms and Dosing Cohorts.

Cohort / Assay	Time Window (h)	No. of Points (N)	Pearson r	Adjusted R2	%AUCextra (%)	Status / Reliability
IV Administration						
ELISA 100 µg	4 – 168	7	-0.881	0.732	45.90%	Unreliable (Radj2<0.80, %AUCextra>20%)
ELISA 50 µg	8 – 168	6	-0.984	0.961	12.30%	Acceptable / Estimable
ELISA 25 µg	72 – 168	3	-0.992	0.968	8.30%	Acceptable / Estimable
LC-MS/MS 100 µg	24 – 168	5	-0.882	0.704	64.70%	Unreliable (Radj2<0.80, %AUCextra>20%)
IM Administration						
ELISA 100 µg	72 – 168	3	-0.913	0.667	28.60%	Unreliable (Radj2<0.80, %AUCextra>20%)
ELISA 50 µg	72 – 168	3	-0.991	0.963	19.70%	Acceptable / Estimable
ELISA 25 µg	48 – 168	4	-0.63	0.095	47.40%	Inadequate fit (Radj2=0.095, NE)
LC-MS/MS 100 µg	48 – 168	4	-0.757	0.358	27.40%	Unreliable (Radj2<0.80, %AUCextra>20%)

Table S2. Linear Regression Parameters and ANCOVA Comparison across Administration Routes and Analytical Platforms.

Comparison / Dataset	Slope (95% CI)	Y-intercept (95% CI)	Goodness of Fit (R^2)	Residual Std Dev (Sy.x)	Runs Test (P-value)	ANCOVA Comparison
A. Method Comparison (LC-MS/MS vs. ELISA)						
• IV Route (100 µg)	0.8158<br>(0.7636 to 0.8679)	6.075<br>(3.734 to 8.416)	0.9898	2.057	0.0047*	—
• IM Route (100 µg)	0.7206<br>(0.3984 to 1.0430)	5.643<br>(0.4559 to 10.83)	0.6644	5.672	0.9225	—
B. Linear Profiles & ANCOVA (IV Delivery)						
• IV LC-MS/MS	-0.1521<br>(-0.3775 to 0.0734)	42.14<br>(29.28 to 55.01)	0.1525	18.72	0.6166	Pooled Slope: -0.1795
• IV ELISA	-0.2070<br>(-0.4758 to 0.0619)	44.84<br>(29.50 to 60.18)	0.19	22.33	0.6166	Pooled Intercept: 43.49
C. Linear Profiles & ANCOVA (IM Delivery)						
• IM LC-MS/MS	0.1109<br>(0.0159 to 0.2059)	11.23<br>(5.809 to 16.65)	0.3504	7.891	0.4126	Pooled Slope: 0.1066
• IM ELISA	0.1023<br>(-0.0145 to 0.2191)	9.319<br>(2.655 to 15.98)	0.2327	9.7	0.0041*	Pooled Intercept: 10.27

*Runs test indicated slight departure from strict linearity, driven by concentration dynamic range / proportional bias.
